# Sweat glucose and GLUT2 expression in atopic dermatitis: Implication for clinical manifestation and treatment

**DOI:** 10.1371/journal.pone.0195960

**Published:** 2018-04-20

**Authors:** Emi Ono, Hiroyuki Murota, Yuki Mori, Yoshichika Yoshioka, Yuko Nomura, Takichi Munetsugu, Hiroo Yokozeki, Ichiro Katayama

**Affiliations:** 1 Department of Dermatology, Course of Integrated Medicine, Graduate School of Medicine, Osaka University, Suita, Osaka, Japan; 2 Biofunctional Imaging Lab, Immunology Frontier Research Center, Osaka University, Suita, Osaka, Japan; 3 Nomura Dermatology Clinic, Yokohama, Kanagawa, Japan; 4 Department of Dermatology, Tokyo Medical and Dental University, Bunkyo-ku, Tokyo, Japan; Universitatsklinikum Hamburg-Eppendorf, GERMANY

## Abstract

Sweat includes active components and metabolites, which are needed to maintain skin homeostasis. Component changes in sweat derived from atopic dermatitis (AD) have been reported. To investigate the influence of sweat components on the pathogenesis of AD, we performed a multifaceted assessment, including nuclear magnetic resonance spectroscopy-based metabolomic analysis, and linked these features to clinical features of AD. Distinctive properties of AD sweat are the quite-variation in protein, anti-microbial peptides and glucose concentrations. pH, sodium, and other salt levels in sweat of AD were comparable to that of healthy subjects. Sweat from AD patients with acute inflammation had a more prominent increase in glucose concentration than sweat from healthy individuals or those with AD with chronic inflammation. Topical glucose application delayed recovery of transepidermal water loss in barrier-disrupted mice. Furthermore, the glucose transporter GLUT2 was highly expressed in the lumen of sweat glands from AD patients. AD patients with chronic inflammation had significantly increased *GLUT2* mRNA expression and near normal sweat glucose levels. Despite the small sample size in our study, we speculate that the increased glucose levels might be affected by AD severity and phenotype. We hope that this report will bring novel insight into the impact of sweat components on the clinical manifestation of AD.

## Introduction

Sweat helps maintain homeostasis in humans [1–3], providing thermoregulation, protection from infection and irritants (e.g., proteases and allergens), and moisturizing effects. These critical functions are mediated by the substances contained in sweat. Sweat contains natural moisturizing factors (e.g., lactate and potassium), anti-microbial factors (e.g., dermcidin, defensins, and cathelicidin), and protease inhibitors (e.g., cysteine protease inhibitor) that form a defensive barrier on the skin surface [4–9]. Moreover, sweat-derived proteases (e.g., kallikreins and kininase) and a selective inhibitor of kallikreins (SPINK6) regulate the skin desquamation process [10, 11]. Therefore, changes in sweat components affect the homeostatic function of skin.

Despite the importance of sweating, patients with atopic dermatitis (AD) believe it has a negative impact on their condition. In a large-scale survey, AD patients thought that sweating from exercise worsened their symptoms [12]. Furthermore, several studies reported that sweat from patients with AD had altered concentrations of antimicrobials, such as dermcidin and soluble IgA [13–15]. In addition, indirect observations indicate that aberrant content of natural moisturizing factors in sweat may be involved in the etiology of atopic dry skin [9, 16]. Therefore, the accumulation of knowledge about sweat components in AD will help to better understand the etiology of AD.

Thus, we compared the components of sweat from healthy subjects and AD patients, using both comprehensive and quantitative strategies, and focused on metabolites in sweat, which are potential biomarkers for various diseases [17]. For the analytical approach, we used nuclear magnetic resonance (NMR) to analyze sweat metabolites as reported previously [18], because NMR can assess sweat components reproducibly and comprehensively. We investigated the biological and metabolomic properties of sweat from healthy subjects and patients with AD to identify biochemical characteristics associated with the negative impacts of sweat in AD patients.

## Results

### Demographic and clinical background of study subjects

Table 1 summarizes the demographic characteristics of the AD patients. The age range was 17–66 years (mean, 37.38), and the male:female gender ratio was 11:10. The SCORing Atopic Dermatitis (SCORAD) scores ranged from 35.2 to 92 (mean, 57.32). Phenotypic characteristics of skin lesions were classified as eczema/exudative papules and lichenification/dermatitis. Cases 3 and 11 were comorbid for erythroderma and prurigo, respectively. The measurements from cases 4 and 7 were obtained from previously preserved sweat samples, as described in the Materials and Methods. Although most AD subjects presented with skin dryness, three subjects (cases 14, 16, and 20) had severe dry skin. Healthy subjects had no skin inflammation or known systemic diseases. Regarding the demographic characteristics of healthy subjects, their ages ranged from 28–84 years (mean, 42), and the male:female gender ratio was 6:4 (S1 Table). For patients with other dermatoses, the age range was 34–78 years (mean, 49), and the male:female gender ratio was 1:5 (S2 Table).

**Table 1:** Properties of sweat from patients with atopic dermatitis.

Case	Age/sex	Onset of AD	SCORAD	Skin symptom	Protein(mg/ml)	pH	Glucose(mg/l)	HbA1c(%)	LL37(ng/ml)	Dermcidin(ng/ml)	β-defensin(pg/ml)	Salt (%)
1*	42/M	early childhood	53.8	Ec/Exd Pap	2.207	6.3	18.9	ND	0.4	0	58	ND
2*	35/F	early childhood	38	Lich/Der	2.416	7.4	2.25	ND	0.4	0	62.5	0.36
3*	44/F	21 yo	70.8	Ec/Exd Pap,erythroderma	3.566	6.3	52.2	ND	0.4	0	60	ND
4*	17/M	early childhood	36.6	Ec/Exd Pap	1.419	7.4	1.8	ND	0.3	0	50	0.39
5*	30/M	early childhood	64.1	Ec/Exd Pap	4.355	5.65	88.2	ND	0.45	0	1000	0.32
6*	29/M	early childhood	45.6	Ec/Exd Pap	1.212	7.8	0.9	4.9	0.9	0	50	ND
7	18/M	17 yo	64.5	Ec/Exd Pap	7.697	7.3	94.5	ND	1.1	0.5	480	ND
8*	27/F	early childhood	36.9	Ec/Exd Pap	2.424	7.9	1.8	ND	0.4	0	55	0.73
9*	38/M	early childhood	61	Ec/Exd Pap	7.157	6.6	135	5.2	0.9	0	750	ND
10	44/M	25 yo	36.9	Lich/Der	ND	ND	2.7	ND	0	0	58	0.33
11*	22/M	early childhood	51.9	Lich/Der, prurigo	ND	ND	10.8	ND	0	0	750	0.26
12*	50/M	early childhood	77	Ec/Exd Pap	7.114	8.27	108	ND	ND	ND	ND	ND
13	42/M	early childhood	35.2	Lich/Der	1.667	ND	2.7	ND	ND	ND	ND	ND
14	30/F	early childhood	72.9	Dry skin, dermatitis	1.662	7.27	76.5	ND	1.2	0	48	0.23
15	66/F	early childhood	65.1	Lichenization	1.212	4.86	1.8	ND	ND	0.5	300	0.1
16	35/F	early childhood	68.5	Dry skin, dermatitis	0.512	5.25	1.8	ND	0.4	0	58	0.09
17	47/F	early childhood	58.7	Lich/Der,dry skin	0.664	4.48	1.8	ND	0.1	0	50	0.07
18	48/F	early childhood	84	Ec/Exd Pap	2.345	5	97.2	ND	0.4	0	300	0.15
19	29/F	early childhood	35.9	Ec/Exd Pap	ND	ND	0.9	ND	0.1	0	60	0.31
20	52/F	early childhood	92	Lich/Der,dry skin	1.323	ND	1.8	ND	ND	ND	ND	0.23
21*	40/M	18 yo	54.5	Lich/Der	ND	ND	2.7	5.6	ND	ND	ND	0.07

Key: M, male; F, female; yo, years old; Ec/Exd Pap, eczema/exudative papules; Lich/Der, lichenification/dermatitis; ND, not done; SCORAD, SCORing Atopic Dermatitis. AD skin samples used in immunohistopathological analysis of GLUT2 (n = 11) were derived from the patients denoted by an asterisk.

### Biological properties of sweat from patients with AD

**Fig 1:**
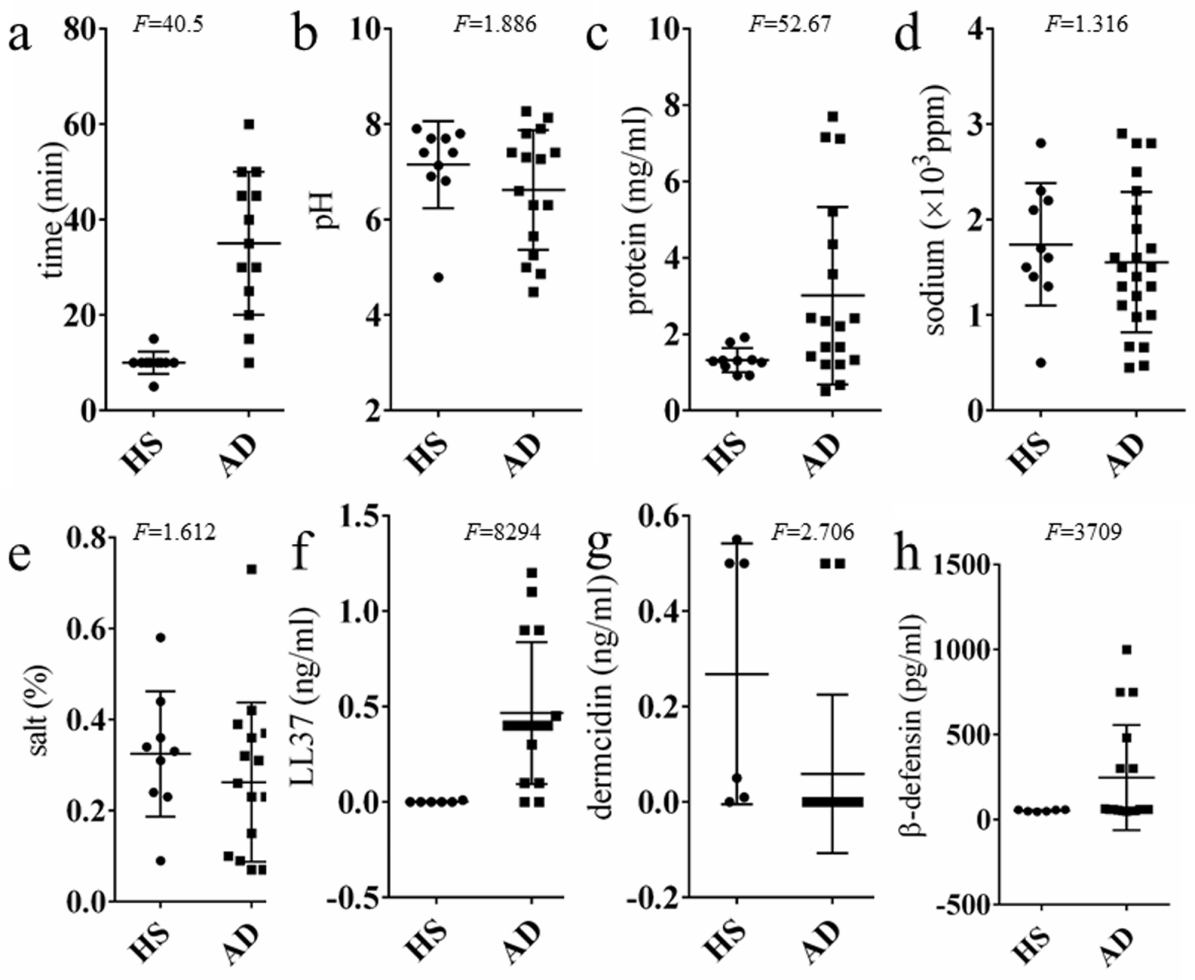
The properties of sweat from patients with atopic dermatitis (AD) and healthy subjects (HS). **(a)** Time required to obtain 5 ml of sweat (HS n = 10, AD n = 13). Analysis of: **(b)** pH (HS n = 10, AD n = 15); **(c)** protein concentration (HS n = 10, AD n = 17); **(d)** sodium concentration (HS n = 10, AD n = 14); and **(e)** other salt content (HS n = 9, AD n = 20). Concentration in sweat of anti-microbial peptides, including: **(f)** LL-37 (HS n = 6, AD n = 16); **(g)** dermcidin (HS n = 6, AD n = 17); and **(h)** β-defensin (HS n = 6, AD n = 17). *F*-test values represent individual variability within the sample.

We collected 5 ml of sweat from the skin of the back for our analyses. Sweat collection took longer for AD patients than for healthy subjects (Fig 1a). Sweat pH values in patients with AD were comparable to those in healthy subjects (Fig 1b). Protein, sodium, and salt concentrations were also assessed. The protein concentration of sweat from patients with AD showed considerably more individual variability compared to healthy subjects (*F* = 52.67(*p*<0.0001)) (Fig 1c). Furthermore, both sodium and salt concentrations showed homoscedasticity between healthy subjects and patients with AD (*F* = 1.316 (*p* = 0.6942), *F* = 1.612 (*p* = 0.5029), respectively) (Fig 1d and 1e). Despite a small sample size, anti-microbial peptide concentrations were measured (Fig 1f, 1g and 1h). Levels of LL37 (the human derivation of cathelicidin) and β-defensin levels also showed greater variance in AD patients than healthy subjects (*F* = 8294 (*p*<0.0001), *F* = 3709 (*p*<0.0001), respectively) (Fig 1f and 1h). However, individual variation of dermcidin was not significantly different between patients with AD and healthy subjects (*F* = 2.706 (*p* = 0.1177)) (Fig 1h).

### NMR-based metabolomic analysis of sweat from patients with AD

**Fig 2:**
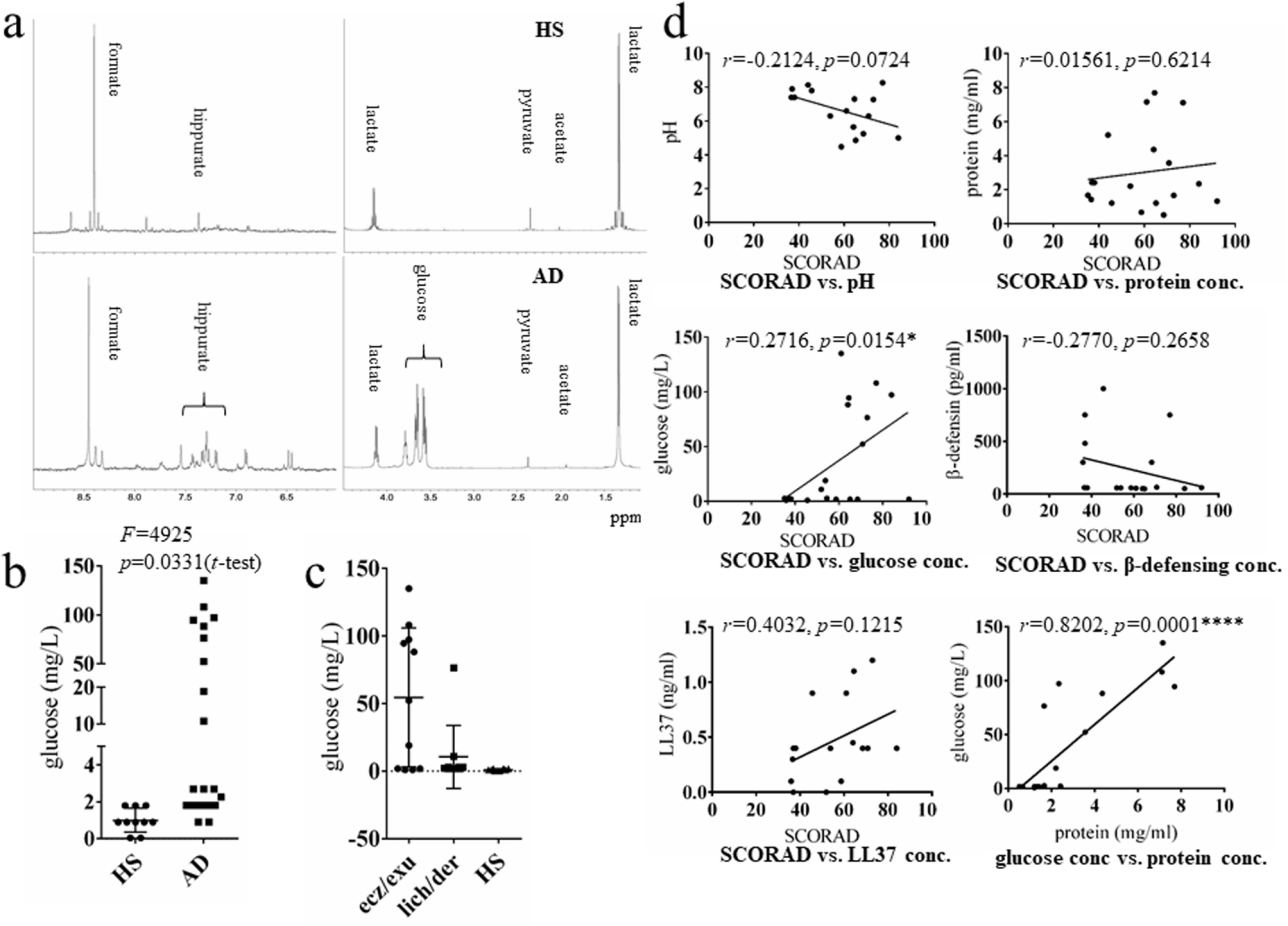
NMR spectra of sweat from patients with AD and HS. **(a)** NMR spectra of the sweat from HS were characterized by intense lactate, pyruvate, and formate signals. A glucose peak was observed in sweat from AD. Glucose concentration was compared between **(b)** HS (n = 10) and AD (n = 21) and **(c)** between AD with eczema/exudative papules (ez/exu, n = 11), AD with lichenification/dermatitis (lich/der, (n = 10) and HS (n = 10). Key: (**p<0.01), unpaired *t*-test. **(d)** Correlation between disease severity (SCORAD) and properties of sweat. Statistical results are presented with Pearson’s correlation coefficients: pH, n = 16; protein concentration, n = 18; glucose concentration, n = 21; β-defensin, n = 18; and LL-37, n = 16.

Metabolic products in sweat were analyzed using NMR. Representative NMR spectra from healthy subjects and patients with AD are shown in Fig 2a. Two peaks, identified as glucose and hippurate, were specific to patients with AD (Fig 2a). Quantitative measurement of glucose concentration revealed that the glucose level was significantly higher (*p* = 0.0331) in sweat from patients with AD (mean±SD: 33.54±45.57, n = 21) compared with healthy subjects (mean±SD: 0.999±0.2053, n = 10) (Fig 2b). Moreover, Glucose concentration was significantly higher in sweat from patients with AD who had eczema/exudative papules, compared with those who had chronic dermatitis/lichenification and healthy subjects (*p*<0.05) (Fig 2c). Moreover, sweat glucose concentration was positively correlated with disease severity (SCORAD) (*r* = 0.2716, *p* = 0.0154) (Fig 2d). Protein concentration, anti-microbial peptide concentration, and pH did not correlate with disease severity (Fig 2d). Sweat glucose concentration was significantly correlated with protein concentration (*p* = .0001). We initially hypothesized that plasma contamination from damaged skin could increase sweat glucose concentration in patients with AD; however, other plasma contamination components were not observed in the NMR spectrum. All subjects included in the study ate lunch at a scheduled time to reduce the likelihood that the time of food ingestion would affect sweat content. Because increased sweat glucose was an unexpected result, serum glucose levels were not measured at the time of sweat collection. HbA1c was measured in cases 6, 9, and 21 (Table 1), but no patients showed abnormally increased HbA1c levels and there was no correlation between HbA1c and sweat glucose. None of the subjects included in the study were diagnosed with diabetes mellitus.

When considering the treatment methods used for our AD subjects (S3 Table), some severe cases treated without topical application of potent corticosteroids (cases 15, 16, and 20) showed comparatively lower sweat glucose levels, whereas many of the AD subjects with high sweat glucose levels had been treated with potent topical corticosteroids.

### Impact of glucose on recovery of damaged stratum corneum in mice

**Fig 3:**
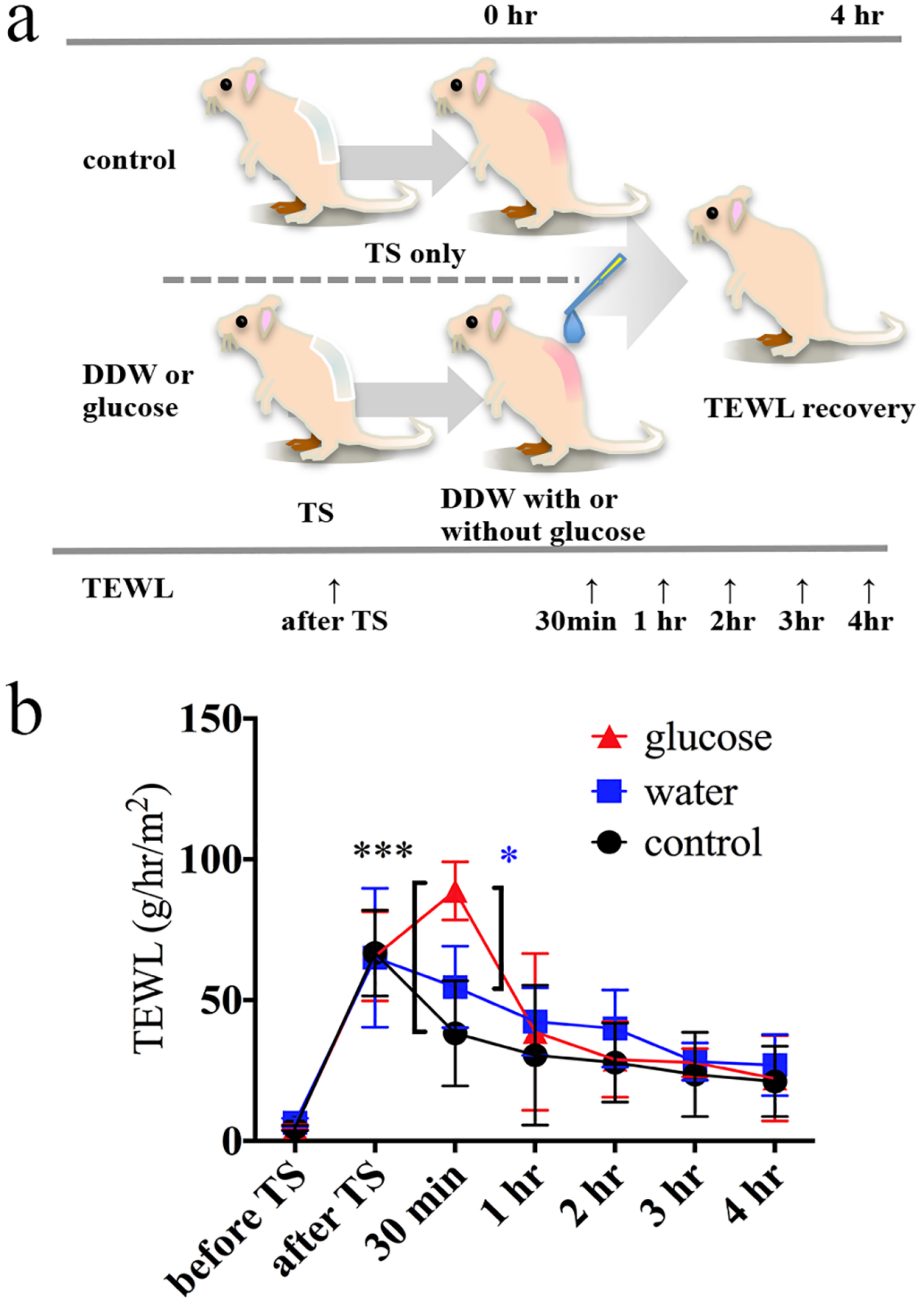
Impact of glucose on recovery of damaged stratum corneum in mice. **(a)** Time course for measuring transepidermal water loss (TEWL) following tape stripping in the barrier disruption mouse model. **(b)** Changes in the TEWL value during the time course. Red triangles represent the glucose-treated group (n = 3), blue squares represent the vehicle-treated group (n = 3), and black circles represent the control group (n = 3). Error bars indicate mean ± SD. Key: (***) *p*<0.001 (glucose vs. control), (*) *p*<0.05 (glucose vs. water), Tukey’s multiple comparison. DDW, distilled deionized water; TS, tape stripping. These results are from one independent experiment; another is shown in S1 Fig.

To investigate the impact of glucose-containing sweat on homeostatic mechanisms, we topically applied a solution of glucose with a concentration (33 mg/l) equivalent to the mean sweat glucose concentration of patients with AD, and investigated its effects on the recovery of damaged stratum corneum in mice. Recovery of the barrier was measured using a transepidermal water loss (TEWL) measurement during the 4 hours after tape stripping (Fig 3a). TEWL values in control and water (vehicle)-treated groups decreased after tape stripping, whereas the TEWL value increased significantly 30 min after tape stripping in the glucose-treated group in two independent experiments (Fig 3b, S1 Fig, and S4 Table). One hour following tape stripping, TEWL values of the glucose-treated group decreased to approximately that of the control group (Fig 3, S1 Fig, and S4 Table). To test whether the osmotic pressure of the glucose solution affected these results, an NaCl solution with the same osmotic pressure as the glucose solution (33 mg/l) was prepared as an osmotic control solution and used in the same protocol. There was no apparent difference in the temporal pattern of TEWL after tape stripping between control or vehicle-treated and osmotic control-treated groups (S2 Fig). These results indicate that glucose in sweat might delay the early phase of recovery of an impaired skin barrier.

### Expression of glucose transporters in sweat glands

**Fig 4:**
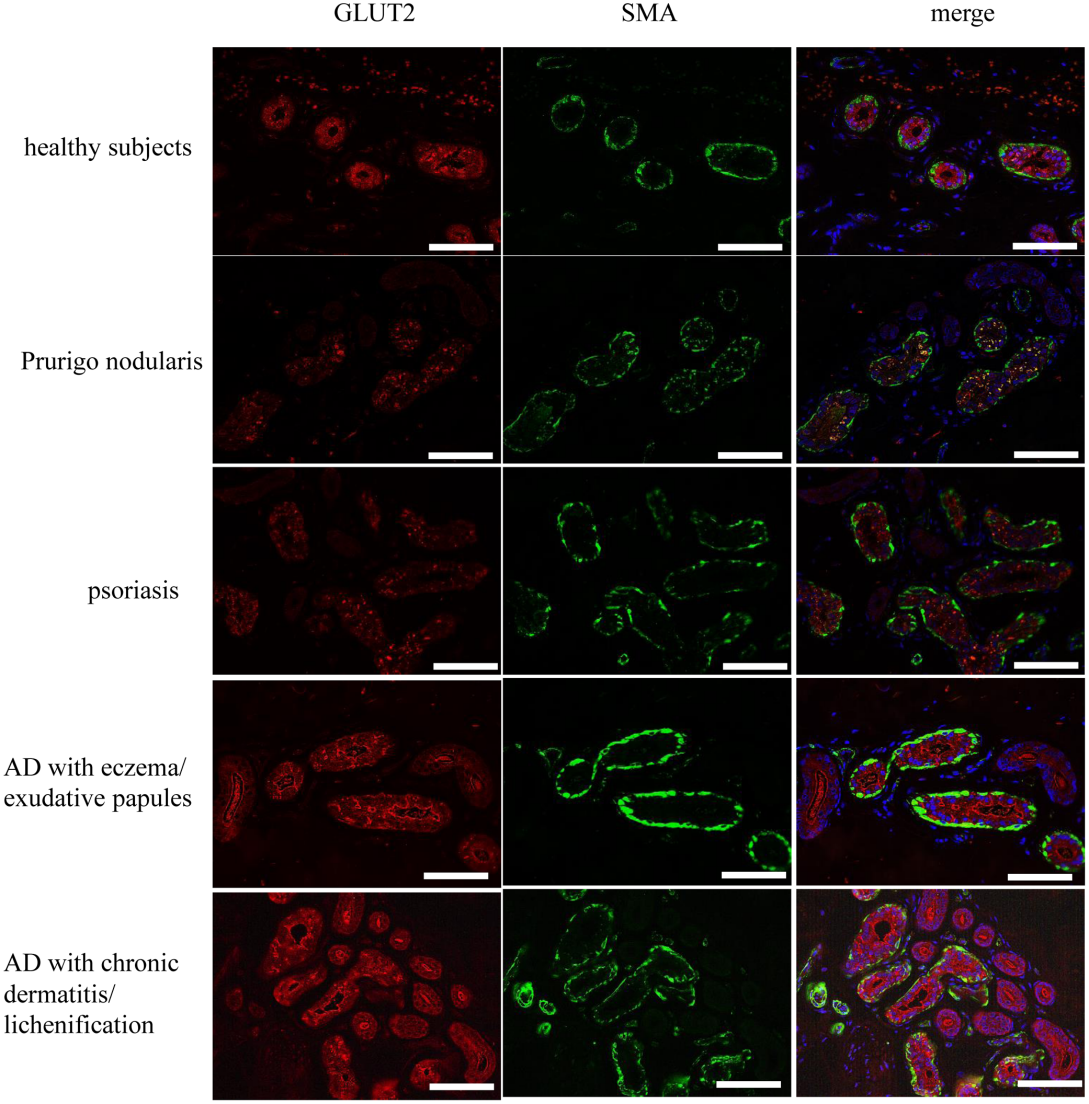
GLUT2 localization in skin from patients with AD. The localization of GLUT2 (red) and smooth muscle actin (SMA; green) in healthy subjects (n = 3), patients with prurigo nodularis (n = 3), psoriasis (n = 3), AD with eczema/exudative papules (n = 8), or AD with dermatitis/lichenification (n = 3) as determined using immunofluorescence. Hoechst33322 was used to stain nuclei (blue). Representative data are presented. Scale bars are 100 μm.

**Fig 5:**
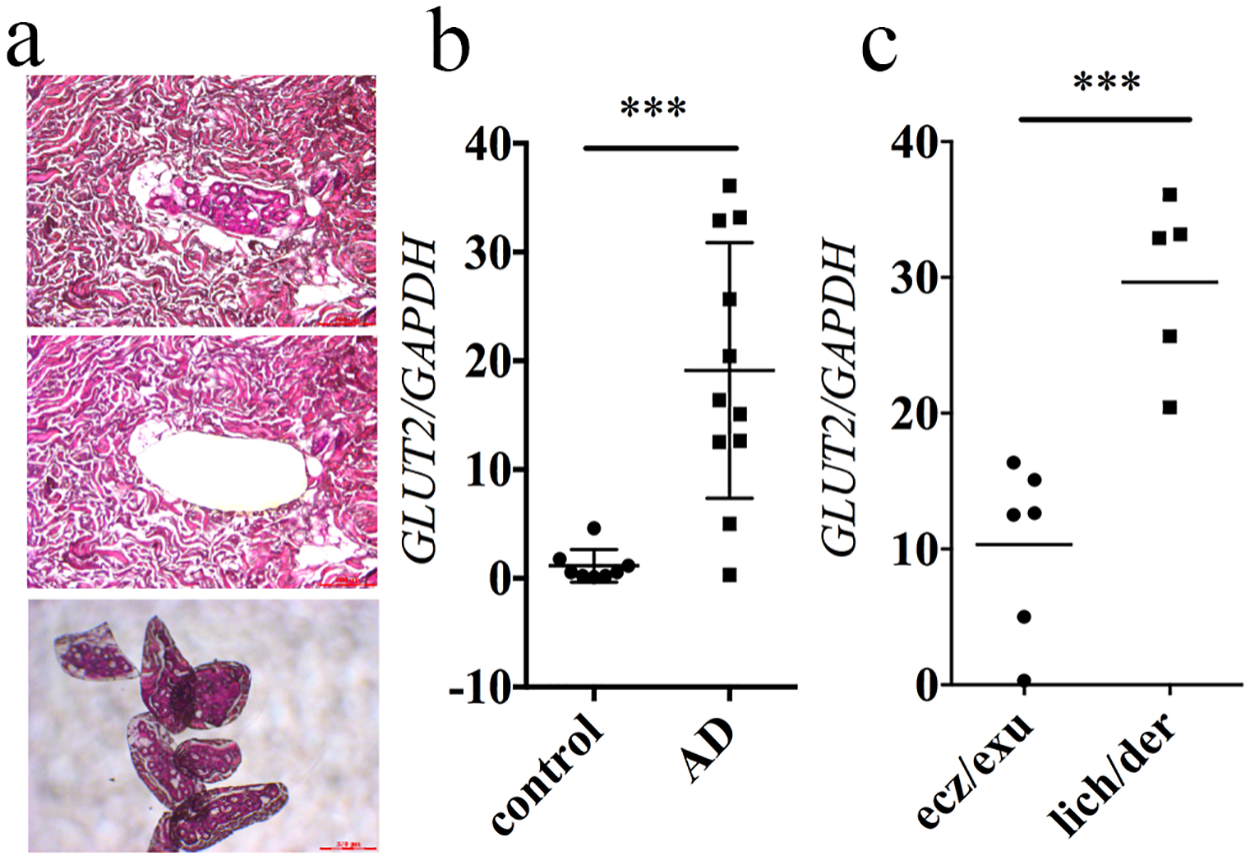
Expression of GLUT2 in sweat glands isolated from human skin. **(a)** Hematoxylin and eosin staining of LMD-harvested sweat glands. **(b)** *GLUT2* mRNA was significantly increased in sweat glands from patients with AD (n = 11) compared with those from HS (control; n = 8). **(C)** *GLUT2* mRNA expression was compared between samples of AD with eczema/exudative papules (n = 6) and AD with dermatitis/lichenification (n = 5). Key: (***) *p*<0.001, unpaired *t*-test.

The expression of both glucose transporters (GLUTs) and sodium/glucose cotransporters (SGLTs) in sweat glands has not been fully investigated. A lack of GLUT3 expression in human sweat glands was confirmed in a previous report [19]. Thus, immunohistochemical staining was performed to investigate the localization of GLUT1, GLUT2, GLUT4, SGLT1, SGLT2, SGLT3, and SGLT4 in sweat glands (Fig 4 and S3 Fig). GLUT2, SGLT3, and SGLT4 were expressed in intact human sweat gland secretory cells as granules in the cytosol that were localized to the basolateral cytoplasmic membrane (Fig 4). Because sodium levels were comparable between sweat from healthy subjects and from AD patients (Fig 1c), we took particular note of the expression of GLUT2. Sweat glands from patients with conditions such as psoriasis and prurigo nodularis displayed a GLUT2 staining pattern similar to that of normal skin (Fig 4 and S4 Fig). Conversely, GLUT2 staining was intense and localized to the luminal cytoplasmic membrane of sweat glands from patients with AD (Fig 4). To quantitatively analyze *GLUT2* expression, real-time PCR was used to assess the level of *GLUT2* mRNA in sweat glands isolated from human skin using laser-capture micro dissection (LMD) (Fig 5a). *GLUT2* mRNA levels increased significantly in sweat glands from patients with AD compared with those from healthy subjects (*p* = 0.0005, unpaired *t*-test) (Fig 5b). Unlike the sweat glucose concentration, *GLUT2* mRNA expression was significantly lower in sweat glands from AD patients with eczema/exudative papules compared with that from those with dermatitis/lichenification (*p* = 0.0007, unpaired *t*-test) (Fig 5c). Thus, *GLUT2* mRNA expression levels do not necessarily correspond to sweat glucose levels, but might be associated with different skin manifestations of AD.

## Discussion

Components of sweat serve an important function in maintenance of homeostasis; therefore, changes in the components of sweat could affect normal skin function. Sweat also can potentially serve as a biological sample for evaluating disease condition. In this study, we investigated the properties of sweat from patients with AD and found that it had different features compared with sweat from healthy subjects.

Firstly, the pH of sweat from AD patients was comparable to, but slightly lower than that from healthy subjects, without statistical significance. To date, the pH of sweat derived from various sudomotor stimuli has stirred up some controversy [20]. Until we performed this study, we expected that an increase in the pH of AD sweat may contribute to barrier dysfunction in AD. Sulzberger et al. observed that patients with AD had increased skin pH, despite the low pH of sweat inside papulovesicles [21]. Sweat pH is controlled by the ratio of bicarbonate ions (HCO_3_^-^) to carbon dioxide (CO_2_), and is affected by sweat rate [2]. Low sweat rate decreases the pH of sweat by allowing reabsorption of HCO_3_^-^ from sweat ducts [2]. The low sweat rate observed in patients with AD could account for the lower sweat pH. However, if this hypothesis were true, sodium and other salt concentrations should also decrease, because sodium and chloride reabsorption in sweat ducts is similarly affected by sweat rate [2]. In this study, although there was the individual variability, concentrations of sodium and other salts in sweat from AD patients were comparable to those from healthy subject sweat. Thus, the altered properties of sweat from AD patients may not be the result of low sweat rate alone.

Recently, sweat components have drawn attention as potential biomarkers for diagnosing and evaluating the severity of certain diseases [9, 18]. Amino acid components in sweat from AD patients, assessed using gas chromatography/time-of-flight mass spectrometry, were reflective of decreased stratum corneum integrity [9]. NMR-based metabolomics studies found that metabolic profiles of sweat were conserved in healthy humans [18]. Our present study revealed that glucose and hippuric acid were distinctive constituents of sweat from AD patients.

Glucose is a known constituent of sweat [2, 22]. Boysen et al. collected sweat using an anaerobic method and reported glucose concentrations of 0.2–0.5 mg/dl at a blood glucose level of 80 mg/dl [22]. Our sweat collection method was aerobic, and the glucose concentrations were lower than those in the Boysen study. Nonetheless, sweat from AD patients largely had higher glucose concentrations than sweat from healthy subjects. As the lactate peak on the NMR spectrum did not differ between sweat from healthy subjects and AD patients (Fig 2), the higher glucose level is likely not due to alterations in the glycolytic system of sweat glands [23]. Sweat glucose concentration positively correlated with AD disease severity, increased in subjects with eczema, and increased moderately in subjects with lichenification/dermatitis. In contrast, protein concentration was not correlated with disease severity, but positively correlated with glucose concentration (Fig 2d). Thus, it seems possible that there might be differences in glucose utilization in the sweat glands of AD patients (i.e., less metabolism by oxidative phosphorylation). Blood contamination due to excoriation could be excluded by comparison with the NMR spectrum of sweat derived from a hematohidrosis patient (case report submitted for publication) and with the NMR spectrum for blood plasma [24].

The causes and mechanisms of increased sweat glucose in AD remain obscure. Although sweat glucose levels in AD subjects correlated significantly with disease severity, some subjects with AD were exceptions to this trend. We identified three severe AD cases with low sweat glucose levels (S3 Table, cases 15, 16, and 20). To our surprise, those cases were treated with only moisturizers or mild corticosteroids, but not with potent topical corticosteroids. This indicated that severe AD subjects could also exhibit low sweat glucose levels in some cases. Furthermore, it could be speculated that topical potent corticosteroid application might affect sweat constituents. To confirm this hypothesis, a larger number of AD subjects treated with or without topical corticosteroids should be recruited. However, one severe AD patient allowed us to collect his sweat before and after therapy; we observed that his increased sweat glucose level decreased as his disease improved (S5 Fig). Therefore, it is still questionable whether topical treatments (e.g. corticosteroids) can affect the components of AD sweat, and their effects may be complex and difficult to interpret. Although the sample size was too small, sweat from patients with other dermatoses, who had used topical corticosteroids on their backs, did not show increased glucose level (data were not shown). Thus, increased glucose in sweat from AD patients might not necessarily coincide with topical corticosteroid treatment.

Topical application of a glucose solution with the same concentration of glucose as that found in sweat from AD patients significantly increased the TEWL value 30 minutes after tape stripping in skin barrier-disrupted mice (Fig 3, S1 Fig, and S4 Table). The aberrantly increased TEWL in glucose-applied mice decreased after one hour, compared to measurements from control- or water (vehicle)-treated mice. Previous articles reported that topical glucose application to barrier-disrupted skin did not affect the recovery of TEWL 1 to 2.5 hours after barrier disruption, consistent with our present study’s results, although those reports did not measure TEWL at earlier time points, such as 30 minutes after applying glucose [25]. Future studies should examine the impact of glucose on the skin microbiome or differential proliferation of epidermal cells.

We also investigated the mechanism by which glucose increased by evaluating glucose transporter expression in sweat glands. GLUTs are facilitative transporters, and SGLTs are sodium-dependent transporters. They are known to play a major role in glucose transport [26]. GLUT1, GLUT2, GLUT3, and GLUT4 expresses mainly in all tissues, hepatocytes/intestine, neurons, and adipose tissue/skeletal muscle, respectively [26]. SGLT1 and SGLT2 are predominantly expressed in the intestine and kidney cortex, respectively, and transport glucose and galactose [26]. There is a functional kinship between the kidney and sweat glands; therefore, we focused on the function of these glucose transporters in sweat glands.

In this study, we investigated GLUT1, 2, and 4 expression in sweat glands. Because sodium concentration was not increased in sweat from AD patients, we focused on the role of GLUT2 in the sweat glands of AD patients. GLUT*2* mRNA expression in sweat glands of AD patients increased significantly compared with sweat glands from healthy subjects. Interestingly, moderately increased *GLUT2* mRNA expression and prominently increased sweat glucose concentration was found in AD patients with eczema or exudative papules, whereas prominently increased *GLUT2* mRNA expression and moderately increased sweat glucose concentration was observed in AD patients with lichenification/chronic dermatitis. It could be speculated that there is a relation between skin manifestation and pattern of GLUT2 expression in sweat gland. Further studies are needed to confirm this hypothesis.

Although the sample size of our study was small, we found that some AD cases showed aberrantly increased sweat glucose. Our findings suggest that this could be related in some way to clinical manifestations, and severity of AD. Thus, we believe that studying the metabolites in sweat will increase our understanding of the pathogenesis of AD.

## Materials and methods

### Study subjects

This study was performed in adherence with the Declaration of Helsinki. This study was approved by the ethical committee of Osaka University Hospital (ID: 13422, 15019). Inpatients with AD (n = 21, Table 1) who fulfilled both the Hanifin and Rajka diagnostic criteria and the Japanese Dermatological Association criteria for AD, healthy subjects (n = 10, age range 28–84, mean 42, male:female 6:4, S1 Table), and inpatients with other dermatoses (n = 6, age range 34–78, male:female = 1:5, S2 Table) were enrolled in this study. Healthy volunteers were recruited by an open recruitment method using posters. All subjects were given precise explanation and briefing paper, and gave informed consent. Previously collected sweat samples from two underage subjects (cases 4 and 7) were also analyzed in this study for the purpose of accumulating subjects from a wide range of backgrounds. Subjects and their guardians gave written consent for the secondary use of these existing samples, which was approved by the institutional ethical committee (ID:17258). Healthy volunteers were recruited using an open recruitment design. AD severity was assessed using SCORAD scoring. Inpatients were given topical treatment (S1 Table) in the morning (approximately 9 AM) and evening (7–8 PM). Meal service was provided to inpatients at approximately 8 AM, 12 PM, and 6 PM.

### Sweat collection

**Fig 6:**
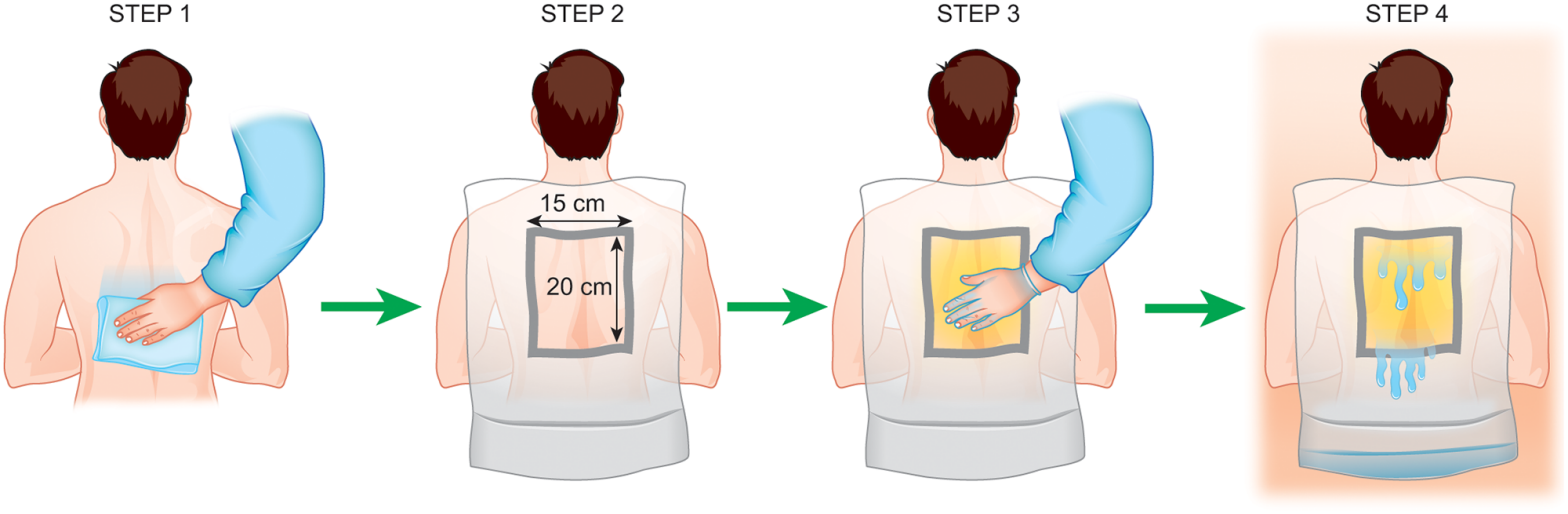
Illustration of the sweat collection procedure. Briefly, the whole back was wiped with tap water (STEP 1), and a drape with a 15×20 cm square hole was applied (STEP 2). Petrolatum was applied to the area in the hole (STEP 3). The bottom of the drape was folded up to create a pocket for fluid collection as subjects bathed in a sauna at 80°C (STEP 4). The sweat that pooled in the drape was collected with a syringe and filtered with a cartridge-type 0.22-μm filter.

All sweat samples used in this study were collected from the backs of subjects in a sauna. Before entering the sauna, the subjects’ backs were wiped with a towel dampened with tap water. A nylon sheet with a square hole (15×20 cm) was fixed to the skin of the back with double-sided tape, and petrolatum was applied evenly to the area inside the hole. Sweat flowed over the surface of the petrolatum, thereby helping to reduce contamination with substances that adhere to the skin surface. The bottom of the nylon sheet was raised to construct a simple pocket to collect sweat. This procedure is illustrated in Fig 6. The subject sat in the sauna for 15–30 minutes, and then sweat was collected with a syringe and filtered with a 0.22-μm syringe filter (Merck Millipore, Darmstadt, Germany) to decrease the contamination of both debris and petrolatum. Sweat collection was performed 2 h after lunch. All subjects were instructed to avoid snacking in the period between lunch and sweat collection, but were allowed to drink water to alleviate dry mouth. Sweat samples previously obtained from cases 4 and 7 were collected to examine an allergy against self-sweat when they were admitted to our hospital on 2014/5/16 and 2013/12/20, respectively. The procedure for sweat collection was identical to that described above. The remaining filtered sweat had been stored at -80°C. Regarding the time required to obtain 5 ml of sweat (Fig 1a), 10 healthy subjects and 13 AD subjects produced 5 ml or more of sweat within 30 minuites of sauna bathing. Other subjects did not yield 5 ml of sweat. If the sweat volume was inadequate to measure all of the evaluation items, we gave priority to NMR spectroscopy and glucose measuerment.

### Assessment of the biological properties of sweat

The pH, sodium concentration, and other salt concentrations were measured with a handheld pH meter ASP-01 (Asahi Bio Med Co Ltd, Tokyo, Japan), sodium ion meter LAQUAtwin B-722 (Horiba, Kyoto, Japan), and salt meter LAQUAtwin B-721 (Horiba), respectively. ELISA assays were performed according to the manufacturers’ instruction to determine dermcidin (CUSBIO BIOTHECH, College Park, MD, USA), β-defensin (Phoenix Pharmaceuticals, Inc., Burlingame, CA, USA), LL-37 (Hycult Bioteck, Uden, Netherlands), and glucose (BioVision, Milpitas, CA, USA) levels.

### NMR spectroscopy

One-dimensional (1D) ^1^H NMR spectra were acquired using a Bruker AVANCE II 500WB spectrometer operating at 499.93 MHz. Collected sweat samples were mixed with 99.9% D_2_O (10% of total volume for each sweat sample) and transferred to standard 5 mm NMR tubes. Time-domain NMR signals were recorded using a single-pulse sequence with the following experimental conditions: water pre-saturation, 2 s; 90° pulse; spectral width, 10 kHz; data points, 32K; repetition time, 11.6 s; accumulation, 2048. Line broadening (3 Hz) was applied prior to the Fourier transformation. NMR signal acquisition and processing were performed using Bruker TopSpin 2.1 software.

### Immunohistochemical staining

Skin sections from patients with AD (n = 11), benign tumors (n = 3), or other dermatoses were assessed immunohistochemically (n = 3 per disease). All patients gave consent for research usage of their redundant skin sample (institutional ethical approval ID: 13422). AD skin samples (n = 11) were derived from the patients denoted by an asterisk in Table 1. The control subjects and those with other dermatoses from whom skin samples were derived from were separate from the subjets enrolled in the sweat study.

Sections (4 μm thick) were incubated overnight at 4°C with anti-GLUT1 (pH 6.0 buffer, 1:200 dilution; cat. no. ab15309; Abcam, Cambridge, UK), anti-GLUT2 (pH 6.0 buffer, 1:300 dilution; cat. no. sd-9117; H-67, Santa Cruz Biotechnology, Santa Cruz, CA, USA), anti-GLUT4 (pH 6.0 buffer, 1:2000 dilution; cat. no. ab48547; Abcam), anti-SGLT1 (pH 9.0 buffer, 1:275 dilution; cat. no. ab14685; Abcam), anti-SGLT2 (pH6.0 buffer, 1:200 dilution; cat. no. ab85626; Abcam), anti-SGLT3 (pH 6.0 buffer, 1:200 dilution; cat. no. 24327-1-AP-150; SGLT3/SLC5A4, Proteintech, Rosemont, IL, USA), anti-SGLT4 (pH 6.0 buffer, 1:100 dilution; cat. no. BMP074; MBL, Nagoya, Japan), or anti-smooth muscle actin (SMA) (pH 6.0 buffer, 1:100 dilution; cat. no. ab15734; Abcam) primary antibodies. As a negative control, rabbit and mouse negative control antibodies were used at the same dilution as the antibodies being compared (cat. no. X0903 and X0931; DAKO). Secondary antibodies included anti-rabbit and anti-goat conjugated to Alexa Fluor 488 (Invitrogen, Carlsbad, CA, USA), and anti-rabbit and anti-mouse antibodies conjugated to Alexa Fluor 555 (Invitrogen). Images of immunolabeled sections were collected with a BZ-8000 microscope (Keyence).

### Evaluation of the impact of glucose in a skin barrier-disrupted mouse model

Animal experiments were performed in accordance with protocols approved by the animal studies committee of Osaka University School of Medicine (ID: 24-114-5). Twelve-week-old female HOSHR1 mice were purchased from CLEA (Osaka, Japan). Mice were maintained in our pathogen-free animal facility. All animal care was in accordance with both the institutional guidelines of Osaka University and the Guidelines for Proper Conduct of Animal Experiments provided by the Science Council of Japan (http://www.scj.go.jp/ja/info/kohyo/pdf/kohyo-20-k16-2e.pdf).

Mouse skin barrier disruption was performed on the back (3×3cm) using a standardized tape-stripping procedure [27]. Mice were anesthetized by intraperitoneal injection of ketamine/xylazine/acepromazine (60 mg/kg, 10 mg/kg, and 2 mg/kg, respectively) to minimize animal suffering and distress. TEWL on the dorsal skin was measured using a Vaposcan^®^ (Asahi Techno Lab. Ltd., Kanagawa, Japan). After tape stripping eight times using SHAMROCK^®^ tape (2 cm × 12.7 m; TOHO, Tokyo Japan), TEWL was measured three times in the same back region per mouse and the average values were calculated.

After tape stripping, 100μl of test solution was applied directly to only the barrier-disrupted skin. Solutions included glucose (33 mg/l in water, which is the mean glucose concentration of sweat from patients with AD) or vehicle (distilled water) and an osmotic pressure control NaCl solution (S2 Fig). The NaCl solution had the same osmotic pressure as the glucose solution (33 mg/l; 0.199 mOsm/Kg) and was prepared by dissolving 0.536 mg of NaCl into 1 l of water (R = 8.31×10^3^ L Pa/K mol, 25°C = 298 K). Subsequently, skin barrier function was measured using TEWL at baseline, immediately after tape stripping, and at 30 min, 1 h, 2 h, 3 h, and 4 h after applying the test solution. In all mice, excess solutions was blotted with Kim-wipes 5 minutes before 30-min TEWL measurement. This examination was performed twice independently. Intraperitoneal injection of pentobarbital sodium (200 mg/kg) was used for euthanasia.

To compare the spread of the glucose solution and water, we applied 25 μl of each solution by pipet to a hydrochromic sheet (PILOT, Japan) that is colored by water (S6 Fig). We did not observe any difference in spreading between the glucose solution and water. We also investigated the change in weight over time of filter paper impregnated with each solution (water, 33 mg/l (0.183 mM) glucose, and controls including 1.83 mM glucose, 18.3 mM glucose, glycerol, and ethanol) to determine the rates of evaporation. Circular paper filters with a radius of 1 cm were impregnated with 50 μl of each solution, and temporal changes in weight were measured. The amount of weight lost was considered the amount of evaporation. The temporal evaporation volume of the glucose solution was comparable to that of water (S7 Fig).

### LMD coupled with real-time PCR

Microdissection was performed using a Leica LMD 7000 (Leica Microsystems, Wetzlar, Germany). Sections (10 μm thick) were sliced from paraffin blocks of skin sections and mounted onto glass slides with a membrane film specific for LMD. After deparaffinization, sections were stained with hematoxylin and eosin and dried at room temperature. Total RNA from homogenized tissues was isolated using the RNeasy FFPE kit (Qiagen, Hilden, Germany) according to the manufacturer’s protocol. The quality of the total RNA was measured using a Nanodrop 2000 (Thermo Scientific, Wilmington, DE, USA). The total RNA was reverse-transcribed to cDNA using the ReverTra Ace qPCR RT Master Mix kit (TOYOBO, Osaka, Japan). Primers for *GLUT2* were generated based on previously published sequences [28]. Real-time PCR reactions were performed using THUNDERBIRD SYBR qPCR Mix (TOYOBO).

### Statistical analysis

Prism 6 software (GraphPad software, La Jolla, CA, USA) was used for statistical analysis. Statistical significance was examined using a Pearson’s correlation coefficient test, an unpaired *t*-test, or a Tukey’s multiple comparison test, as appropriate. Bars in dot plots represent the mean ± SD. Statistical significance was defined as *p*<0.05 for the appropriate adjusted *p* value.

## Supporting information

S1 Fig The reproducibility of Fig 3b experiments. Changes in the TEWL value during the time course of the tape stripping were measured independently for Fig 3b to confirm the reproducibility of our experiments. Red triangles represent the glucose-treated group (n = 3); blue squares represent the vehicle-treated group (n = 3); and black circles represent the control group (n = 3). Key: error bars indicate mean ± SD; (***) p<0.001 (glucose vs. control at 30 min), (*) p<0.05 (glucose vs. water at 30 min, glucose vs. control at 1 h), Tukey’s multiple comparison; DDW, distilled deionized water; TS, tape stripping.
(PDF)

S2 Fig Impact of osmolarity control solution application on barrier recovery. NaCl solution with the same osmotic pressure as the glucose solution (33 mg/l; 0.199mOsm/Kg) was used in the same experiment as that in Fig 3. n = 3 per group.
(PDF)

S3 Fig Immunohistochemical staining of GLUT1, -2, -4 and SGLT1–4 in kidney and skin specimens. GLUT1 and -4 and SGLT1 and -2 were not expressed in human sweat glands, but GLUT2 and SGLT3 and -4 were observed. Scale bar indicates 100 μm. Positive control for GLUTs and SGLT1-4 was kidney tissue, for GLUT1 was red blood cells, and for GLUT4 was muscle tissue. Negative control staining for GLUT2 in kidney was performed using rabbit immunoglobin. Bottom right: green, SMA; red, SGLT4; blue, nuclei.
(TIFF)

S4 Fig GLUT2 localization in skin from patients with inflammatory skin diseases. GLUT2 localization in skin specimens from patients with seborrheic keratosis, prurigo nodularis, erythroderma, mycosis fungoides (MF), and pigmented nevus was investigated by immunofluorescenece staining. Three cases are shown per condition. Scale bar indicates 100 μm. Green: SMA, red: GLUT2, blue: nuclei.
(TIFF)

S5 Fig SCORAD score and glucose level before and after treatment. (a) Clinical photograph of patient before treatment. (b) Clinical photograph of patient after treatment. Therapeutic intervention demonstrated improved symptoms. (c) SCORAD score and glucose level before and after treatment. Sweat glucose levels were decreased according to the attenuation of disease severity.
(PDF)

S6 Fig Spreading of glucose solution. The area of spreading of the solutions tested in Fig 3 and S2 Fig were measured using a water-colorimetric hydrochromic sheet. Each solution was applied in triplicate. There was no apparent difference in the measured spread of the glucose solution and vehicle (water).
(PDF)

S7 Fig Evaporation rate of glucose solution. To compare the evaporation rate between glucose solution and its vehicle, temporal changes in the weight of filter paper impregnated with each solution (water, glucose (0.183 mM, 1.83 mM, and 18.3 mM), glycerol, and ethanol) was measured. The concentration of 0.183 mM corresponds to the 33 mg/l tested in Fig 3 and S2 Fig. The amount of weight loss was considered the amount of evaporation. N = 3, graph indicates mean ± SD.
(PDF)

S1 Table Properties of sweat from healthy subjects. (PDF)

S2 Table Properties of sweat from patients with other dermatoses. (PDF)

S3 Table Treatment of the AD patients. (PDF)

S4 Table Concrete data of the transepidermal water loss (TEWL) values in Fig 3b (1st) and S1 Fig (2nd)*. (PDF)
