# Supplementary material for: Sweat glucose and GLUT2 expression in atopic dermatitis: Implication for clinical manifestation and treatment
Source: PLoS One. 2018 Apr 20;13(4):e0195960. doi: 10.1371/journal.pone.0195960 (PMC5909908; doi:10.1371/journal.pone.0195960)
Supplement: S1 Fig — Changes in the TEWL value during the time course of the tape stripping were measured independently for Fig 3b to confirm the reproducibility of our experiments. Red triangles represent the glucose-treated group (n = 3); blue squares represent the vehicle-treated group (n = 3); and black circles represent the control group (n = 3). Key: error bars indicate mean ± SD; (***) p<0.001 (glucose vs. control at 30 min), (*) p<0.05 (glucose vs. water at 30 min, glucose vs. control at 1 h), Tukey’s multiple comparison; DDW, distilled deionized water; TS, tape stripping. (PDF) [file pone.0195960.s001.pdf]

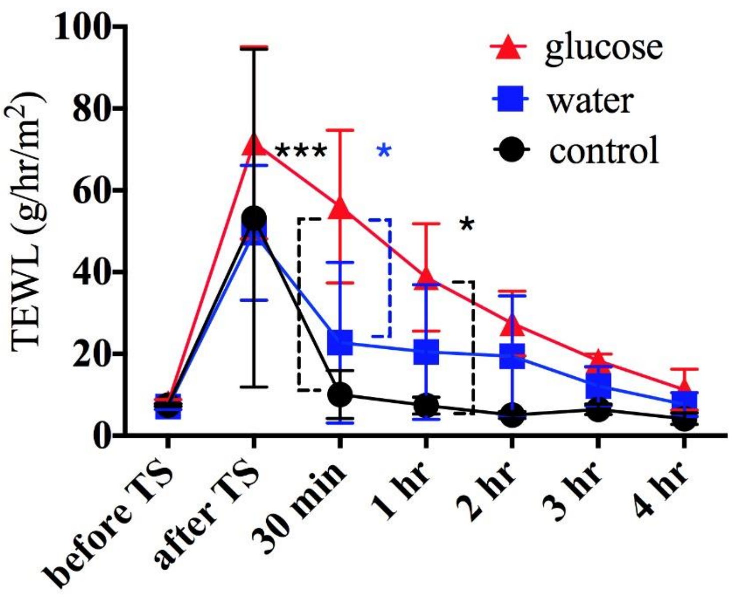

S1 Fig. The reproducibility of Fig 3b experiments.

Changes in the TEWL value during the time course of the tape stripping were measured independently for Fig 3b to confirm the reproducibility of our experiments. Red triangles represent the glucose-treated group (n=3); blue squares represent the vehicle-treated group (n=3); and black circles represent the control group (n=3). Key: error bars indicate mean ± SD; Tukey's multiple comparison, ***$p<0.001$ (glucose vs. control at 30 min), *$p<0.05$ (glucose vs. water at 30 min, glucose vs. control at 1 h); DDW, distilled deionized water; TS, tape stripping.
