# Supplementary material for: Sweat glucose and GLUT2 expression in atopic dermatitis: Implication for clinical manifestation and treatment
Source: PLoS One. 2018 Apr 20;13(4):e0195960. doi: 10.1371/journal.pone.0195960 (PMC5909908; doi:10.1371/journal.pone.0195960)
Supplement: S2 Fig — NaCl solution with the same osmotic pressure as the glucose solution (33 mg/l; 0.199mOsm/Kg) was used in the same experiment as that in Fig 3. n = 3 per group. (PDF) [file pone.0195960.s002.pdf]

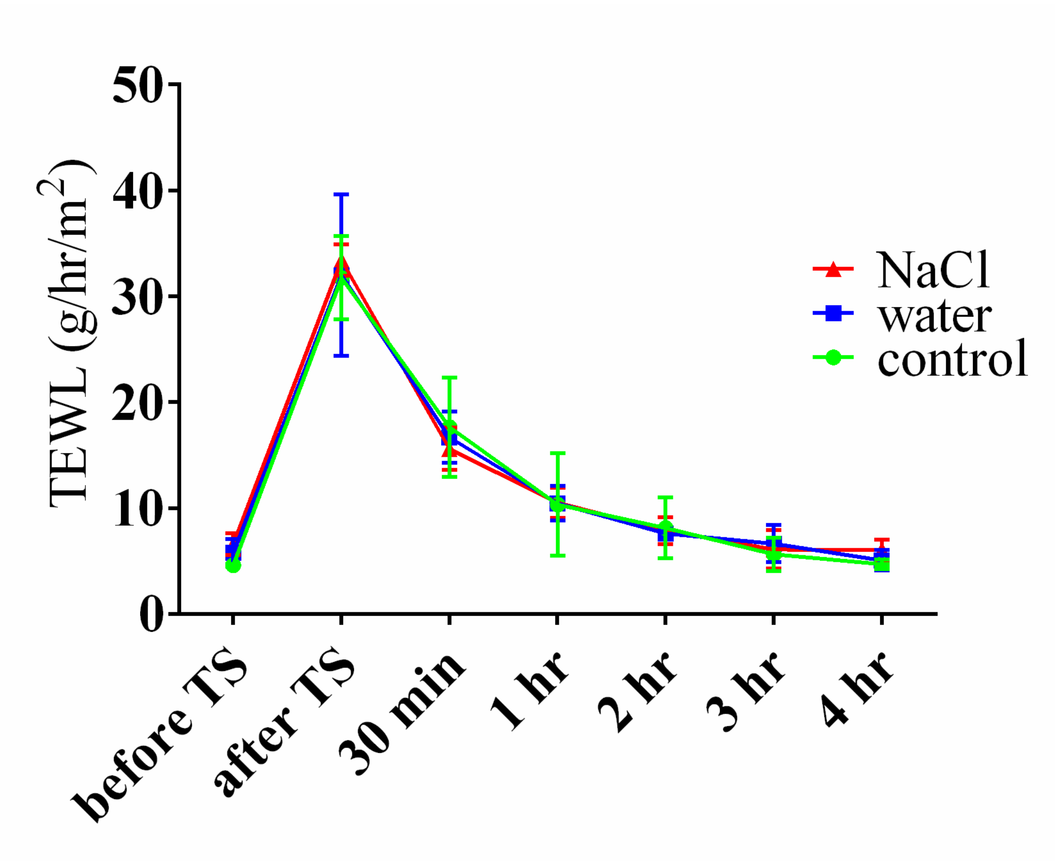

S2 Fig. Impact of osmolarity control solution application on barrier recovery.

NaCl solution with the same osmotic pressure as the glucose solution (33 mg/l; 0.199mOsm/Kg) was used in the same experiment as that in Fig3. n=3 per group.
