# Supplementary figures and images for: Sweat glucose and GLUT2 expression in atopic dermatitis: Implication for clinical manifestation and treatment
Source: PLoS One. 2018 Apr 20;13(4):e0195960. doi: 10.1371/journal.pone.0195960 (PMC5909908; doi:10.1371/journal.pone.0195960)

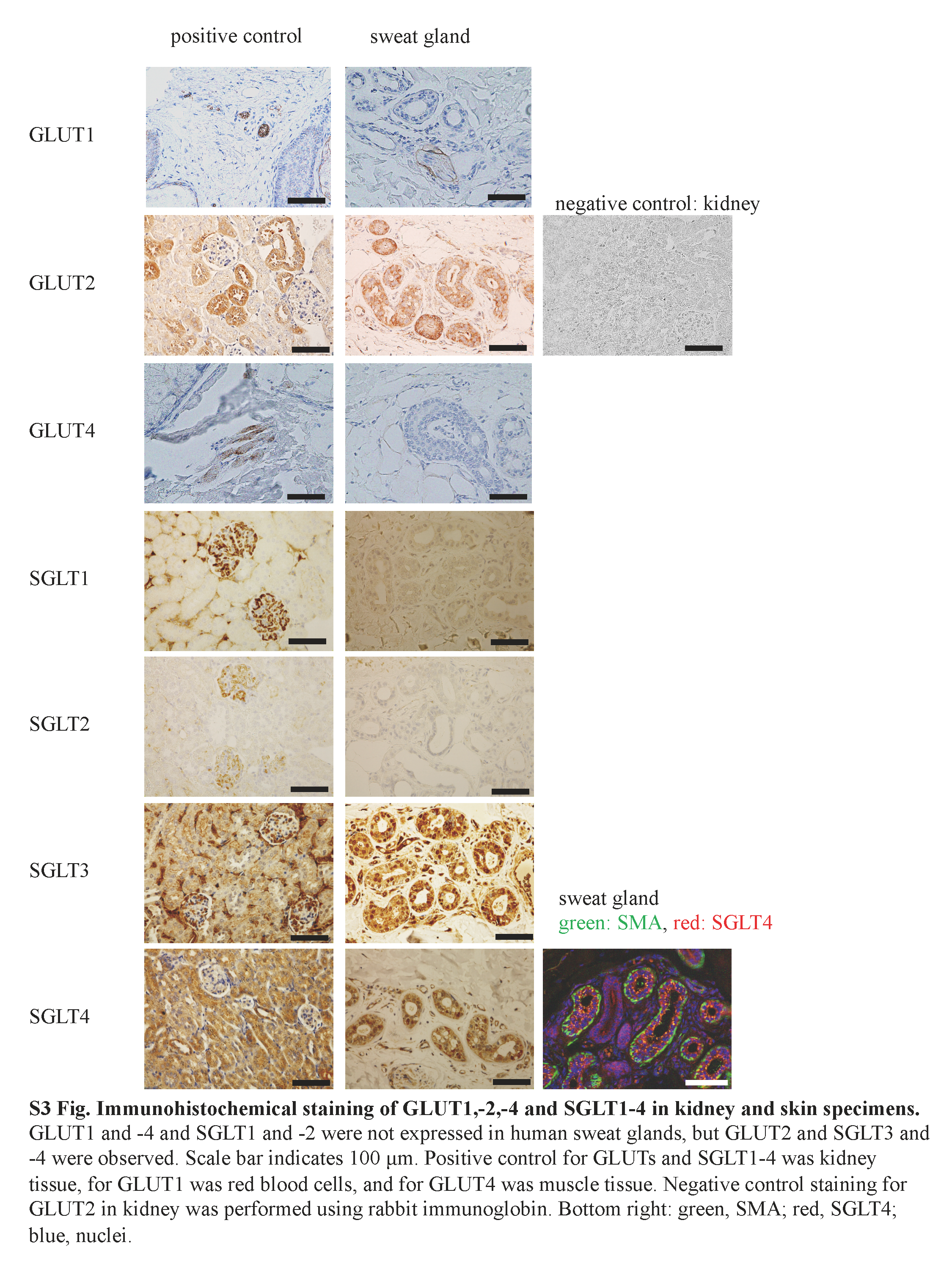

Supplement: S3 Fig — GLUT1 and -4 and SGLT1 and -2 were not expressed in human sweat glands, but GLUT2 and SGLT3 and -4 were observed. Scale bar indicates 100 μm. Positive control for GLUTs and SGLT1-4 was kidney tissue, for GLUT1 was red blood cells, and for GLUT4 was muscle tissue. Negative control staining for GLUT2 in kidney was performed using rabbit immunoglobin. Bottom right: green, SMA; red, SGLT4; blue, nuclei. (TIFF) [file pone.0195960.s003.tiff]

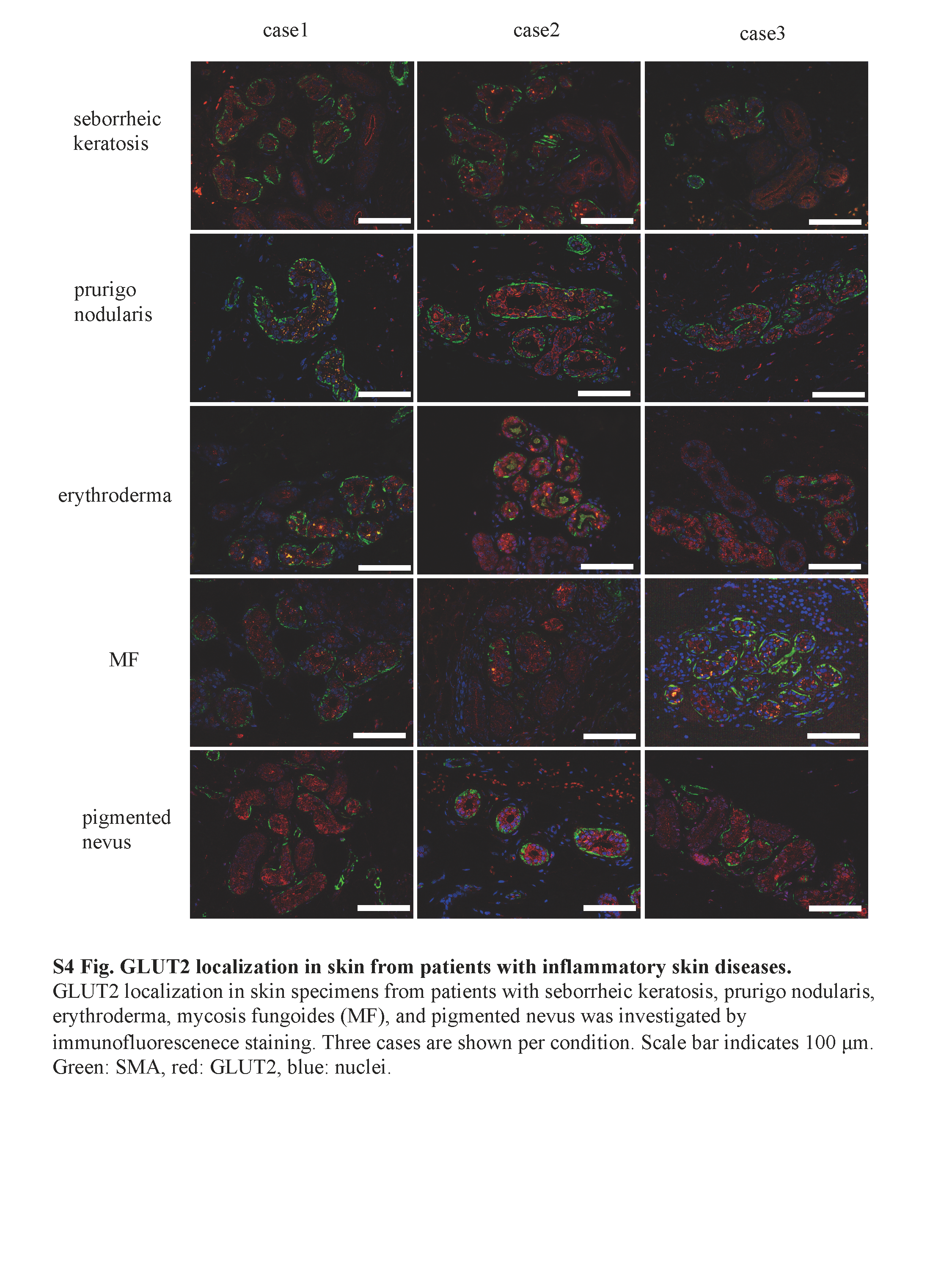

Supplement: S4 Fig — GLUT2 localization in skin specimens from patients with seborrheic keratosis, prurigo nodularis, erythroderma, mycosis fungoides (MF), and pigmented nevus was investigated by immunofluorescenece staining. Three cases are shown per condition. Scale bar indicates 100 μm. Green: SMA, red: GLUT2, blue: nuclei. (TIFF) [file pone.0195960.s004.tiff]
