# Supplementary material for: Sweat glucose and GLUT2 expression in atopic dermatitis: Implication for clinical manifestation and treatment
Source: PLoS One. 2018 Apr 20;13(4):e0195960. doi: 10.1371/journal.pone.0195960 (PMC5909908; doi:10.1371/journal.pone.0195960)
Supplement: S5 Fig — (a) Clinical photograph of patient before treatment. (b) Clinical photograph of patient after treatment. Therapeutic intervention demonstrated improved symptoms. (c) SCORAD score and glucose level before and after treatment. Sweat glucose levels were decreased according to the attenuation of disease severity. (PDF) [file pone.0195960.s005.pdf]

a

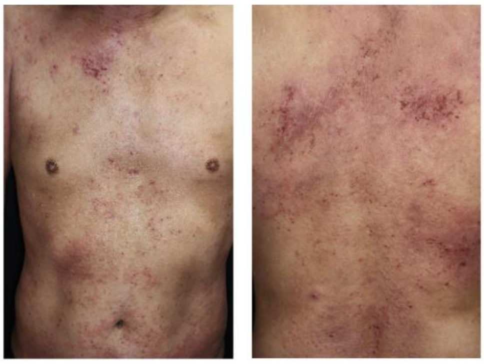

b

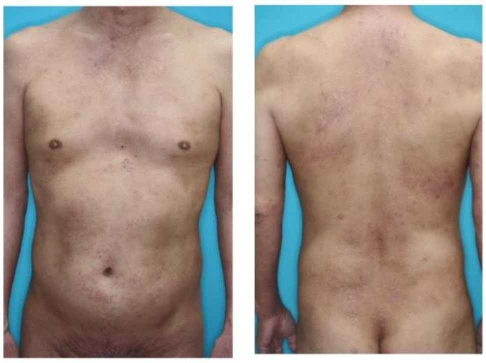

c

	Before treatment	After treatment
**SCORAD score**	77	21
**Glucose (mg/L)**	108	27

S5 Fig. SCORAD score and glucose level before and after treatment.

(a) Clinical photograph of patient before treatment. (b) Clinical photograph of patient after treatment. Therapeutic intervention demonstrated improved symptoms. (c) SCORAD score and glucose level before and after treatment. Sweat glucose levels were decreased according to the attenuation of disease severity.
