# Supplementary material for: Sweat glucose and GLUT2 expression in atopic dermatitis: Implication for clinical manifestation and treatment
Source: PLoS One. 2018 Apr 20;13(4):e0195960. doi: 10.1371/journal.pone.0195960 (PMC5909908; doi:10.1371/journal.pone.0195960)
Supplement: S6 Fig — The area of spreading of the solutions tested in Fig 3 and S2 Fig were measured using a water-colorimetric hydrochromic sheet. Each solution was applied in triplicate. There was no apparent difference in the measured spread of the glucose solution and vehicle (water). (PDF) [file pone.0195960.s006.pdf]

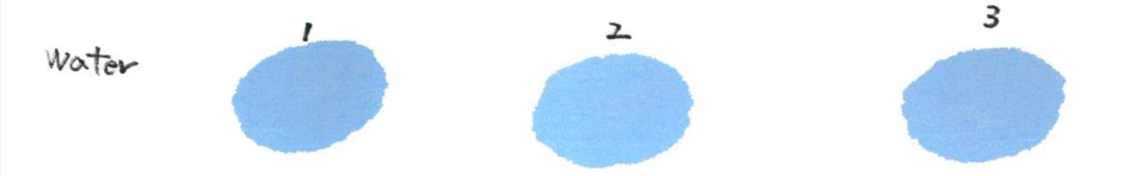

water	2.86×1.63	3.06×2.1	3.06×2.22

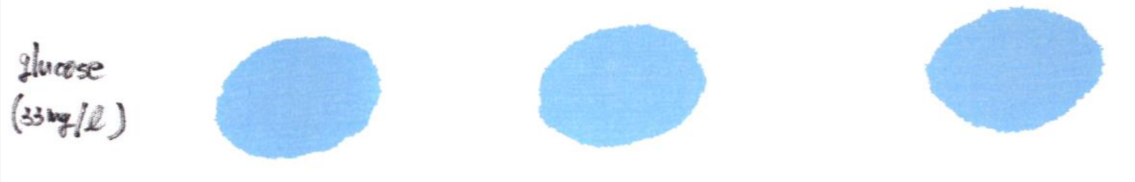

glucose (33mg/l)	3.04×1.98	3.06×2.15	3.07×2.05

S6 Fig. Spreading of glucose solution

The area of spreading of the solutions tested in Fig 3 and S2 Fig were measured using a water-colorimetric hydrochromic sheet. Each solution was applied in triplicate. There was no apparent difference in the measured spread of the glucose solution and vehicle (water).
