# Supplementary material for: Sweat glucose and GLUT2 expression in atopic dermatitis: Implication for clinical manifestation and treatment
Source: PLoS One. 2018 Apr 20;13(4):e0195960. doi: 10.1371/journal.pone.0195960 (PMC5909908; doi:10.1371/journal.pone.0195960)
Supplement: S7 Fig — To compare the evaporation rate between glucose solution and its vehicle, temporal changes in the weight of filter paper impregnated with each solution (water, glucose (0.183 mM, 1.83 mM, and 18.3 mM), glycerol, and ethanol) was measured. The concentration of 0.183 mM corresponds to the 33 mg/l tested in Fig 3 and S2 Fig. The amount of weight loss was considered the amount of evaporation. N = 3, graph indicates mean ± SD. (PDF) [file pone.0195960.s007.pdf]

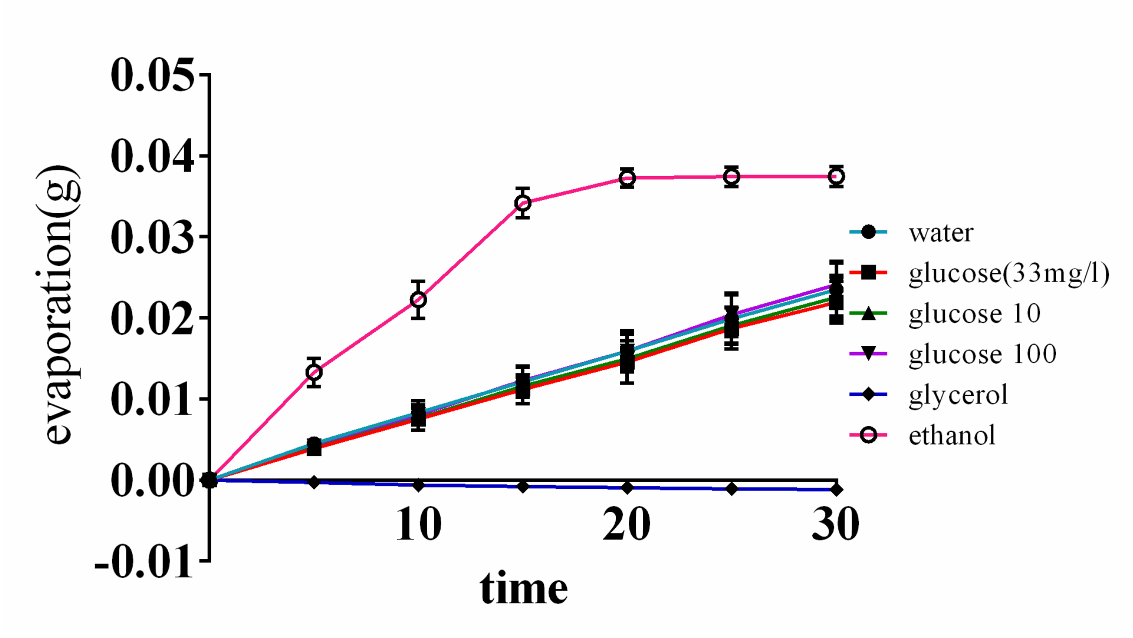

S7 Fig Evaporation rate of glucose solution

To compare the evaporation rate between glucose solution and its vehicle, temporal changes in the weight of filter paper impregnated with each solution (water (n=3), glucose (0.183 mM (n=3), 1.83 mM (n=3), and 18.3 mM (n=3)), glycerol (n=3), and ethanol (n=3)) was measured. The concentration of 0.183 mM corresponds to the 33 mg/l tested in Fig 3 and S2 Fig. The amount of weight loss was considered the amount of evaporation. N=3, graph indicates mean ± SD.
