# Supplementary material for: Sweat glucose and GLUT2 expression in atopic dermatitis: Implication for clinical manifestation and treatment
Source: PLoS One. 2018 Apr 20;13(4):e0195960. doi: 10.1371/journal.pone.0195960 (PMC5909908; doi:10.1371/journal.pone.0195960)
Supplement: S1 Table — (PDF) [file pone.0195960.s008.pdf]

S1 Table. Properties of sweat from healthy subjects

Case	Age/ sex	pH	Glucose (mg/l)	LL37 (ng/ml)	Dermcidin (ng/ml)	β-defensin (pg/ml)	Protein (ng/ml)	Sodium (ppm)	Salt (%)
1	44/M	6.9	0.9	0	0.5	46	0.9	2300	0.58
2	36/M	7.8	0.045	0.01	0.5	49	0.917	1400	0.36
3	28/M	7.7	0.9	0	0.55	56	1.259	1700	0.44
4	28/M	7.92	0.9	0	0.01	58	1.914	2800	0.33
5	35/M	7.43	1.8	0	0.05	57	1.786	2100	0.34
6	38/M	7.66	1.8	0	0	49	1.156	2200	ND
7	32/F	4.79	0.9	ND	ND	ND	1.287	500	0.09
8	35/F	7.4	0.045	ND	ND	ND	1.304	1500	0.31
9	61/F	7.13	0.9	ND	ND	ND	1.327	1600	0.24
10	84/F	6.81	1.8	ND	ND	ND	1.309	1300	0.23

*Key: M, male; F, female; ND, not done
