# Supplementary material for: Sweat glucose and GLUT2 expression in atopic dermatitis: Implication for clinical manifestation and treatment
Source: PLoS One. 2018 Apr 20;13(4):e0195960. doi: 10.1371/journal.pone.0195960 (PMC5909908; doi:10.1371/journal.pone.0195960)
Supplement: S2 Table — (PDF) [file pone.0195960.s009.pdf]

S2 Table. Properties of sweat from patients with other dermatoses*

Case	Age/ sex	Diagnosis	pH	Glucose (mg/l)	LL37 (ng/ml)	Dermcidin (ng/ml)	β-defensin (pg/ml)	Protein (ng/ml)	Sodium (ppm)	Salt (%)
1	34/F	Systemic contact dermatitis	4.50	0.9	0.4	0	300	0.163	430	0.08
2	55/F	Chronic urticaria	5.53	0.9	0.4	0.2	0	1.007	1300	0.19
3	38/F	Asteatotic eczema	7.58	1.8	ND	ND	ND	2.231	1900	0.29
4	49/F	Chronic urticaria	7.04	0.9	ND	ND	ND	4.427	2600	0.39
5	40/F	Palmoplantar pustulosis	5.52	0.045	ND	ND	ND	1.014	1300	0.2
6	78/M	Prurigo nodularis	ND	0.9	0	0	0	ND	1300	0.2

*Key: M, male; F, female; ND, not done
