# Supplementary material for: Sweat glucose and GLUT2 expression in atopic dermatitis: Implication for clinical manifestation and treatment
Source: PLoS One. 2018 Apr 20;13(4):e0195960. doi: 10.1371/journal.pone.0195960 (PMC5909908; doi:10.1371/journal.pone.0195960)
Supplement: S3 Table — (PDF) [file pone.0195960.s010.pdf]

## S3 Table. Treatment of the AD patients

| case | Glucose (mg/l) | SCORAD | Topical treatment | | Oral medicine |
|---|---|---|---|---|---|
| | | | Face | Body | |
| 1 | 18.9 | 53.8 | Mild corticosteroid | Very strong corticosteroid | (-) |
| 2 | 2.25 | 38 | Mild corticosteroid | Very strong corticosteroid | (-) |
| 3 | 52.2 | 70.8 | Mild corticosteroid | Very strong corticosteroid | Antihistamine |
| 4 | 1.8 | 36.6 | Mild corticosteroid | Strong corticosteroid | Antihistamine |
| 5 | 88.2 | 64.1 | Tacrolimus | Very strong corticosteroid | Antihistamine |
| 6 | 0.9 | 45.6 | Mild corticosteroid | Very strong corticosteroid | Antihistamine |
| 7 | 94.5 | 64.5 | Mild corticosteroid | Very strong corticosteroid | Antihistamine |
| 8 | 1.8 | 36.9 | Mild corticosteroid | Strong corticosteroid | Antihistamine |
| 9 | 135 | 61 | Ketoconazole | Very strong corticosteroid | Antihistamine |
| 10 | 2.7 | 36.9 | Mild corticosteroid | Very strong corticosteroid | Antihistamine |
| 11 | 10.8 | 51.9 | Tacrolimus | Very strong corticosteroid | Antihistamine |
| 12 | 108 | 77 | Tacrolimus | Very strong corticosteroid | Antihistamine |
| 13 | 2.7 | 35.2 | Mild corticosteroid | Very strong corticosteroid | Antihistamine |
| 14 | 76.5 | 72.9 | Moisturizer | Very strong corticosteroid | Antihistamine |
| 15 | 1.8 | 65.1 | Tacrolimus | Mild corticosteroid | Antihistamine |
| 16 | 1.8 | 68.5 | Moisturizer | Moisturizer | Antihistamine |
| 17 | 1.8 | 58.7 | Moisturizer | Strong corticosteroid | Antihistamine |
| 18 | 97.2 | 84 | Tacrolimus | Strong corticosteroid | Antihistamine |
| 19 | 0.9 | 35.9 | Tacrolimus | Strong corticosteroid | Antihistamine |
| 20 | 1.8 | 92 | Moisturizer | Moisturizer | Antihistamine |
| 21 | 2.7 | 54.5 | Mild corticosteroid | Strong corticosteroid | Antihistamine |

*Key: SCORAD, SCORing Atopic Dermatitis.
