# Supplementary material for: Sweat glucose and GLUT2 expression in atopic dermatitis: Implication for clinical manifestation and treatment
Source: PLoS One. 2018 Apr 20;13(4):e0195960. doi: 10.1371/journal.pone.0195960 (PMC5909908; doi:10.1371/journal.pone.0195960)
Supplement: S4 Table — (PDF) [file pone.0195960.s011.pdf]

S4 Table. Concrete data of the transepidermal water loss (TEWL) values in Fig 3b (1st) and S1 Fig (2nd)*

Time related to tape-stripping (TS) treatment		Control (n=3)	Water (n=3)	Glucose (n=3)
Before TS	1st	4.6±0.95	6.5±1.6	4.9±0.9
	2nd	7.5±0.31	7.1±0.74	8.0±0.78
After TS	1st	66.7±15.2	65±24.7	65.6±15.8
	2nd	53.2±41.29	49.6±16.49	71.6±23.51
30 min post TS	1st	38.2±18.7	54.7±14.5	88.8±10.4
	2nd	10.1±5.8	22.8±19.64	56.0±18.67
1 h post TS	1st	30.5±24.8	42.4±12	38.7±27.9
	2nd	7.37±2.07	20.47±16.45	38.7±13.11
2 h post TS	1st	27.8±14	39.9±13.6	28.9±13.4
	2nd	5.07±0.85	19.4±14.74	27.43±7.9
3 h post TS	1st	23.6±15	28.2±6.6	27.8±5
	2nd	6.43±	12.13±4.8	18.37±1.6
4 h post TS	1st	21.1±12.5	27±10.9	22.2±15.2
	2nd	4.17±1.39	7.63±2.92	11.27±5

***Values are expressed as mean**±SD (g/h/m^2).
